# Age-Specific Composition and Predicted Function of Gut Microbiota in Plateau Pikas (*Ochotona curzoniae*)

**DOI:** 10.3390/biology15020144

**Published:** 2026-01-14

**Authors:** Hui Han, Yongbing Yang, Xiaojia Zhu, Migmar Wangdwei, Le Yang

**Affiliations:** 1 Key Laboratory of Biodiversity and Environment on the Qinghai-Tibetan Plateau, Ministry of Education, Xizang University, Lhasa 850000, China; hanhui1005@126.com; 2 Key Laboratory of Biological Resources and Biosafety, Institute of Plateau Biology Research of Xizang Autonomous Region, Lhasa 850000, China; 18270292589@139.com; 3 College of Life Sciences, Shaanxi Normal University, Xi’an 710119, China; zhuxj2018@snnu.edu.cn

**Keywords:** age stage, gut microbiota, metagenomic sequencing, plateau pika, Qinghai–Tibet Plateau

## Abstract

Gut microbes play a crucial role in regulating physiological processes such as host energy metabolism, nutrient absorption, and environmental adaptation. The predicted functions of gut microbes can be influenced by many factors, both extrinsic and intrinsic to the hosts. The plateau pika is a key species in the alpine ecosystem of the Qinghai–Tibet Plateau. Previous research on the plateau pika primarily examined how extrinsic factors affected its gut microbiota. However, studies on intrinsic factors are scarce. Here, we used live-trapping to capture plateau pikas and collect cecum contents. Using metagenomic sequencing of cecum content samples, we characterized and compared the gut microbial composition and predicted function of plateau pika in adult (*n* = 9) and juvenile (*n* = 9) populations. The results indicated that Bacillota and Bacteroidete were the major bacterial phyla. The core gut microbial genera were the same, but the relative abundance of *Oscillospira* in juveniles was significantly lower than that in adults. The changes in the proportion of cellulose-degradation-related bacterial communities in juveniles suggest that they tend to choose low-fiber diets. In this study, we found no significant differences in the gut microbial composition and diversity, KEGG level 1 metabolic pathways, or CAZy class level between adult and juvenile plateau pikas. In total, the composition and predicted functions of cecal microorganisms in juvenile and adult male plateau pikas were not different. Regarding KEGG level 2 metabolic pathways, the juvenile group had a higher relative abundance of metabolic pathways for cofactors and vitamins, terpenoids, and polyketides, whereas the adult group had a higher relative abundance of energy metabolism. However, the resulting differences remain unclear. Therefore, future research should validate the above findings on a broader spatio-temporal scale and conduct cross-species comparisons to construct a microbial ecological framework for the health management of plateau wild animals.

## Simple Summary

The community of microbes living in the gut is essential for an animal’s health, helping with digestion and adapting to its environment. For the plateau pika, a small mammal native to the high-altitude Qinghai–Tibet Plateau, we wanted to understand if these gut microbes change as the animal ages. During growth and development, the diversity and composition of intestinal microbiota change. Understanding the impact of age on gut microbiota is crucial for further ecological research and conservation management. We compared the gut microbes of adult and juvenile pikas using metagenomic sequencing. We found that the overall types of microbes and predicted functions were very similar between adults and juveniles. However, the changes in the proportion of cellulose-degradation-related bacterial communities in juveniles suggest that they tend to choose low-fiber diets. There were three metabolic pathways with significant differences in the KEGG secondary metabolic pathways. This study, however, had limitations that constrained the generalizability of the results. In the future, more in-depth studies are needed to verify the observed trends and clarify the main drivers of age-related microbial variation in wild herbivores.

## 1. Introduction

The mammalian gut microbiota, encompassing diverse bacterial, fungi, archaea, and virus taxa, plays a pivotal role in host health, regulation nutrient digestion, energy metabolism, development, and immune defense [1,2,3,4]. In natural ecosystems, investigating the composition and predicted functions of the gut microbiota in wild animals will enhance our understanding of gut microbial resources and the mechanism of host–microbiota interactions [5]. Previous research showed that vertebrate gut microbiota composition and function are influenced by synergistic effects of both extrinsic (e.g., diet, attitude, season) and intrinsic factors (e.g., host genetics, life history) [6,7,8,9,10,11,12,13,14,15,16].

Among intrinsic factors, the life history of a species is the result of natural selection, influenced by multiple factors, and can reflect the species’ strategies for adapting to its living environment [17]. During growth and development, the gastrointestinal tract morphology, diet, etc., lead to changes in the diversity and composition of gut microbiota [18,19]. Microbial colonization and growth enhance the resilience and robustness of the microbial community as organisms age [20]. Understanding the impact of age on gut microbiota is crucial for further ecological research and conservation management. Studies on various wild mammals, such as Siberian flying squirrels (*Pteromys volans*) [21], yellow-bellied marmots (*Marmota flaviventer*) [22], and rhesus macaques (*Macaca mulatta*) [23], have consistently revealed significant age-related shifts in gut microbial diversity, composition, and immune-related functions. Overall, gut microbiomes change significantly with age [24]. However, the composition and predicted function of gut microbiota across different life stages in wild animals remain underexplored, particularly on the Qinghai–Tibet Plateau.

The Qinghai–Tibet Plateau, known as the Roof of the World and the Third Pole of the Earth, has an average elevation exceeding 4000 m [25]. This encompasses China’s largest alpine meadow ecosystem, covering approximately 35% of the plateau’s total area. This rangeland has abundant natural resources, which possess significant ecological value and unique environmental characteristics [26,27]. As an endemic and keystone species on the Qinghai–Tibet Plateau, the plateau pika increases plant diversity and improves soil structure. Meanwhile, it drives key ecological processes through behaviors such as foraging and burrowing, playing an irreplaceable role in maintaining the structure and function of the alpine meadow ecosystem [28,29,30]. Research studies on the gut microbiota of plateau pikas mainly focused on factors such as diet [31], season [16,32], altitude [33], and gut region [34]. However, the influence of host life history, specifically age, on its gut microbial ecology constitutes a significant knowledge gap.

In one study, the growth and development of plateau pikas were divided into four stages by age in days: neonatal (1–10 days), juvenile (10–30 days), subadult (30–65 days), and adult (>65 days). During early life stages, individuals weighed less than 80 g. Adult pikas had a minimum weight of 110 g, an approximate maximum weight of 230 g, and an average weight of approximately 150 g [35]. Sampling was conducted during the peak breeding period of plateau pikas. The captured female individuals were in estrus, pregnancy, and lactation periods, which would significantly alter body weight and gut microbial characteristics. Considering the above factors, female individuals were not included in this study. Due to the living characteristics of plateau pikas, the newborns live in burrows, so individuals in the neonatal period could not be obtained. Individuals over 65 days old had mature sexual organs and were capable of reproduction. In this study, based on the critical body weight values of juveniles and adults recorded in the historical literature and whether the testes of males had descended, the captured plateau pika individuals were divided into juveniles and adults. Focusing on these two age stages, metagenomic sequencing technology was used to investigate the species composition and predicted functions of cecal microorganisms in plateau pikas in different age stages. We addressed one key question: whether the composition and predicted functions of cecal microorganisms in juvenile and adult male plateau pikas vary with different age stages. This study aims to preliminarily explore the dynamic changes in the cecal microbial community and predicted functions of plateau pikas in different age stages, providing a research basis for the subsequent protection and health management of plateau wild animals.

## 2. Materials and Methods

### 2.1. Study Area

Samples were collected in Chundui Township, Lhünzhub County, Lhasa City, Tibet Autonomous Region, China, at an altitude of 4077 m (29°55′41.72″ N, 90°57′45.55″ E) above sea level. The Lhasa region boasts rich wild flora and fauna resources [32], providing a unique and ecologically diverse habitat for wildlife, including the plateau pika. The dominant plant species in the region include *Kobresia pygmaea* and *Kobresia littledalei*, alongside *Potentilla anserina*, *Potentilla eriocarpa*, *Lamiophlomis rotata*, *Taraxacum tibetanum*, *Lancea tibetica*, *Androsace tapete*, *Leontopodium pusillum*, *Saussurea* sp., and *Rhodiola* sp. [36], which coexist with other potential taxa adapted to the high-altitude environment.

The area provides habitat for a variety of rare and endangered animals, including the Dhole (*Cuon alpinus*), Brown Bear (*Ursus arctos*), Eurasian Lynx (*Lynx lynx*), Snow Leopard (*Panthera uncia*), Alpine Musk Deer (*Moschus chrysogaster*), White-lipped Deer (*Cervus albirostris*), Bharal (*Pseudois nayaur*), Himalayan Vulture (*Gyps himalayensis*), Tibetan Snowcock (*Tetraogallus tibetanus*), Tibetan Eared Pheasant (Crossoptilon harmani), Black-necked Crane (*Grus nigricollis*) [37], and other wildlife. During field observations, several predator species, such as the Saker Falcon (*Falco cherrug*), Common Kestrel (*Falco tinnunculus*), Upland Buzzard (*Buteo hemilasius*), Tibetan Fox (*Vulpes ferrilata*), and Red Fox (*Vulpes vulpes*), were seen near the plateau pika habitat.

### 2.2. Cecum Contents Sample Collection

In May 2024, researchers used live traps to capture plateau pikas in the study area. The pikas were euthanized via cervical dislocation [38]. All juvenile plateau pikas were capable of independent movement, often observed exploring their surroundings or foraging near the burrow entrance. Ethics approval was obtained from the Institute of Plateau Biology Research of Xizang Autonomous Region ethics committee (ethical approval no.: 20231007). Three sampling points were randomly selected at the sampling site, and 6 pikas were captured at each sampling point, including 3 juveniles and 3 adults, totaling 18 individuals (9 adults and 9 juveniles).

Researchers distinguished juveniles from adults based on body weight. Juveniles weighed an average of 50.36 g ± 17.50 g (min: 28.5 g; max: 71.2 g), while adults had an average weight of 159.57 g ± 14.70 g (min: 136.5 g; max: 183.3 g). Detailed weight data are summarized in Supplementary Table S1. Simultaneously, juveniles and adults were distinguished based on reproductive organs. The reproductive organs of juvenile plateau pikas were underdeveloped. Adult plateau pikas were in the breeding season and exhibited obvious characteristics, such as descended testes in males and open vaginas in females. Considering that pregnancy might affect the gut microbiota composition of plateau pikas, male individuals were selected as adult subjects in this experiment. See Supplementary Table S1 for more details of the sample selection. Dissections were performed on a sterile table, and cecum contents were collected and transferred to 10 mL cryotubes. The samples were then transported in liquid nitrogen and stored at −80 °C [39]. The samples were transported on dry ice to Shanghai Majorbio Bio-Pharm Technology Co., Ltd. (Shanghai, China) for subsequent sequencing analysis. The sequencing platform was Illumina NovaSeq X Plus (Illumina Inc., San Diego, CA, USA), based on the PE150 sequencing strategy, with a sequencing depth of 6G.

### 2.3. Metagenomic Data Processing

#### 2.3.1. DNA Extraction

A total of 0.2 g of cecum content material was used to extract genomic DNA using the FastPure Stool DNA Isolation Kit (Magnetic bead) (MJYH, Shanghai, China), following the manufacturer’s instructions. Negative DNA extraction controls and negative controls for sequencing were processed simultaneously. The concentration and purity of the extracted DNA were assessed with the SynergyHTX (Agilent Technologies, Santa Clara, CA, USA) and NanoDrop2000 (Thermo Scientific, Santa Clara, CA, USA), respectively. The quality of the DNA extract was verified using a 1% agarose gel.

#### 2.3.2. Metagenomic Sequencing

DNA extracts were fragmented to an average size of approximately 400 bp using the Covaris M220 (Gene Company Limited, HongKong, China) for paired-end library construction. The paired-end library was constructed using NEXTFLEX Rapid DNA-Seq (Bioo Scientific, Austin, TX, USA). Paired-end sequencing was performed on the NovaSeq X Plus (Illumina, San Diego, CA, USA) at Majorbio Bio-Pharm Technology Co., Ltd. (Shanghai, China) utilizing the NovaSeq X Series 25B Reagent Kit, following the manufacturer’s instructions (www.illumina.com, accessed on 24 July 2024).

#### 2.3.3. Gene Prediction, Taxonomy, and Functional Annotation

The data were analyzed using the free online Majorbio Cloud Platform (www.majorbio.com, accessed on 2 August 2024). The raw sequencing reads underwent trimming to remove adapters, and low-quality reads (length < 50 bp or with an average quality value < 20) were eliminated using fastp [40] (https://github.com/OpenGene/fastp, version 0.20.0, accessed on 17 August 2024). The reads were aligned to the plateau pika’s genome with BWA [41] (http://bio-bwa.sourceforge.net, version 0.7.9a, accessed on 20 August 2024) and any hits associated with the reads and their paired reads were then excluded.

The quality-filtered data were assembled using MEGAHIT [42] (https://github.com/voutcn/megahit, version 1.1.2, accessed on 8 September 2024). Contigs with a length of 300 bp or more were selected as the final assembly results. Open reading frames (ORFs) from each assembled contig were predicted using Prodigal [43] (https://github.com/hyattpd/Prodigal, version 2.6.3, accessed on 12 September 2024) and ORF lengths of 100 bp or more were retrieved.

A non-redundant gene catalog was created using CD-HIT [44] (https://github.com/weizhongli/cdhit/, version 4.6.1, accessed on 18 September 2024) with 90% sequence identity and 90% coverage. Gene abundance in a specific sample was estimated using SOAPaligner [45] (http://soap.genomics.org.cn, version 2.21, accessed on 29 September 2024) with 95% identity.

#### 2.3.4. Species and Functional Annotation

Representative sequences of the non-redundant gene catalog were aligned with the NR database (NR20230830) (https://ftp.ncbi.nlm.nih.gov/blast/db/FASTA/, version 20200604, accessed on 8 October 2024) with an e-value cutoff of 1 × 10^−5^ using Diamond [46] (https://github.com/bbuchfink/diamond, version 2.0.13, accessed on 11 October 2024). The KEGG annotation was conducted using Diamond against the Kyoto Encyclopedia of Genes and Genomes database (https://www.genome.jp/kegg/, accessed on 13 October 2024) [47] with an e-value cutoff of 1 × 10^−5^. Annotation of carbohydrate-active enzymes was conducted using hmmer (http://www.hmmer.org/, version 3.1b2, accessed on 18 October 2024) against the CAZy database (http://www.cazy.org/, accessed on 20 October 2024) [48] with an e-value cutoff of 1 × 10^−5^.

### 2.4. Statistical Analysis

Percentage stacking diagrams were generated using R to show the relative abundance of KEGG level 1 and 2 metabolic pathways and bacterial communities at the phylum, family, and genus levels [49]. To detect the core microbiome, 80% prevalence and the lowest detection of 0.01 was used [50]. Differences in the core microbiome, KEGG level 1 and 2 metabolic pathways, and CAZy class level between age groups were tested using the Wilcoxon rank-sum test for non-pairwise comparisons. Beta diversity was assessed using the Adonis test and the Bray–Curtis distance and visualized by principal coordinate analysis (PCoA) [51]. Alpha diversity indices (Simpson index, Pielou index, and Shannon index) were calculated using the alpha function from the vegan package. The resulting data were tested for normality and homogeneity of variance. When the data satisfied the assumptions of normality and homogeneity of variance, we used an independent-sample *t*-test to assess intergroup differences. When the assumptions were not met, we applied the Wilcoxon rank-sum test [52]. All *p*-values were corrected for multiple comparisons using FDR correction. Statistical significance levels are indicated as follows: * *p*  < 0.05; ** *p*  < 0.01; *** *p*  < 0.001; **** *p*  < 0.0001.

## 3. Results

### 3.1. Metagenomic Sequencing Data and Microbial Community Analysis for the Juvenile and Adult Plateau Pikas

We performed metagenomic sequencing on 18 samples and obtained 817,695,694 raw reads. After quality control, we retained 810,867,906 high-quality reads, with an average of 45,048,217 reads per sample. After quality control, a total of 626,469,724 optimized reads were obtained after removing the host genome, with an average of 34,803,540 optimized reads, 79.39% ± 4.56% (mean ± SD) of the raw reads. We then assembled the reads and generated 9,719,160 contigs. Detailed assembly statistics are summarized in Supplementary Table S2, Figures S1 and S2. From these contigs, we constructed a nonredundant gene catalog and performed microbial taxonomic annotation. For downstream analyses, we excluded archaea, fungi, and viruses. The final annotated bacterial dataset comprised 22,248 species spanning 3780 genera, 888 families, 429 orders, 252 classes, and 158 phyla.

**Figure 1:**
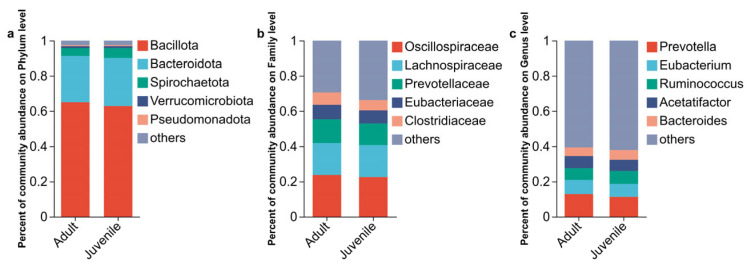
Composition analysis of the cecum microbiome between two groups. The top 5 phyla (**a**), the top 5 families (**b**), and the top 5 genera (**c**).

This study examined the differences in microbial community composition between juvenile and adult plateau pikas at the phylum, family, and genus levels. The most abundant bacterial phyla were Bacillota (65.03% ± 4.82% for adults and 62.83% ± 5.98% for juveniles), Bacteroidota (26.25% ± 3.71% for adults and 27.29% ± 5.74% for juveniles), Spirochaetota (4.58% ± 1.79% for adults and 5.72% ± 4.09% for juveniles), and Verrucomicrobiota (1.06% ± 0.21% for adults and 1.11% ± 0.68% for juveniles) (Figure 1a). At the family level, the cecum microbiota of the plateau pika was dominated by Oscillospiraceae (23.64% ± 4.53% for adults and 22.46% ± 2.61% for juveniles), Lachnospiraceae (18.12% ± 3.58% for adults and 18.15% ± 4.23% for juveniles), Prevotellaceae (13.57% ± 3.77% for adults and 12.29% ± 3.34% for juveniles), and Eubacteriaceae (8.20% ± 1.79% for adults and 7.45% ± 2.60% for juveniles) (Figure 1b). At the genus level, *Prevotella* (12.77% ± 3.71% for adults and 11.29% ± 3.13% for juveniles), *Eubacterium* (8.10% ± 1.80% for adults and 7.36% ± 2.60% for juveniles), *Acetatifactor* (6.80% ± 2.40% for adults and 6.34% ± 2.19% for juveniles), and *Ruminococcus* (6.71% ± 1.27% for adults and 7.30% ± 1.56% for juveniles) were the predominant genera of the cecum microbiota in the two groups (Figure 1c).

**Figure 2:**
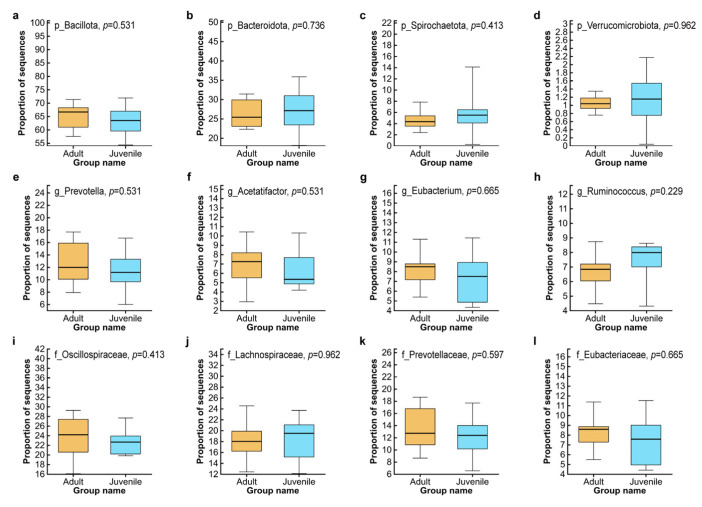
Box plots of the differences in major bacteria at different taxonomic levels between two groups. Phylum level (**a**–**d**), family level (**e**–**h**), and genus level (**i**–**l**).

Through the Wilcoxon rank-sum test, we found that there was no significant difference in the composition of the major cecum microbiota at the phylum level, family level, and genus level between two groups (Figure 2, *p* > 0.05).

### 3.2. Core Microbiome of Juvenile and Adult Plateau Pikas

The core microbiome identified in the study comprised 4 phyla, 9 families, and 17 genera (Table 1). The core cecum microbiota groups were the same between adult and juvenile plateau pikas. Among the four core phyla, Bacillota exhibited the highest abundance in both age groups. At the genus level, *Prevotella* was the most dominant. Through Wilcoxon rank-sum test, we found that the relative abundance of *Oscillibacter* in adults was significantly higher than that in juveniles (*p* < 0.05).

### 3.3. Cecum Microbiota Diversity in Plateau Pikas of Different Age Groups

**Figure 3:**
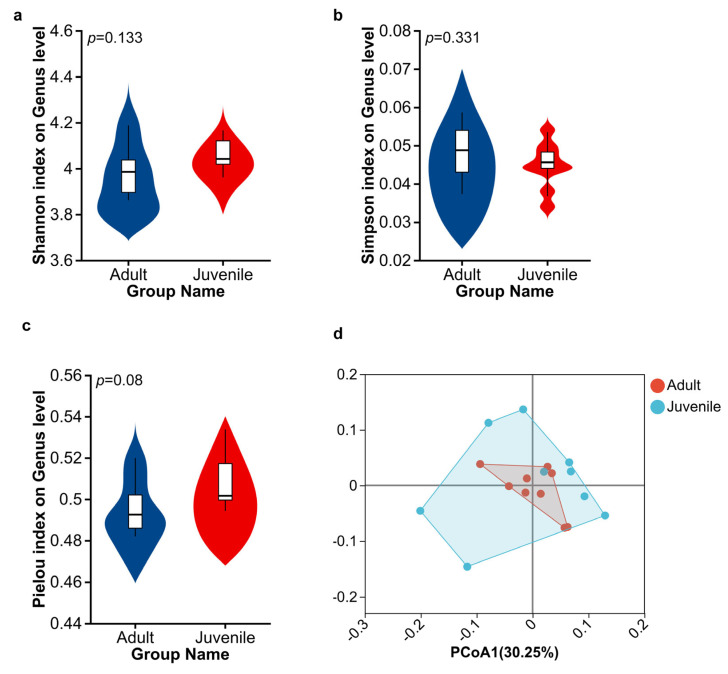
Diversity analysis of microbiome in adult group and juvenile group. (**a**) Shannon index statistics. (**b**) Simpson index statistics. (**c**) Pielou index statistics. (**d**) PCoA of samples in two groups (PCoA1 = 30.25%; PCoA2 = 19.94%).

To investigate the differences in cecum microbial diversity between adult and juvenile plateau pika groups, we calculated genus-level alpha diversity using the Pielou, Shannon, and Simpson indices. Among them, the Shannon index (4.00 ± 0.12 for adults and 4.06 ± 0.08 for juveniles) and Pielou index (0.50 ± 0.01 for adults and 0.51 ± 0.02 for juveniles) of juveniles were higher than those of adults, while the Simpson index (0.048 ± 0.01 for adults and 0.045 ± 0.01 for juveniles) of juveniles was lower than that of adults. However, the differences did not reach a significant level (*p* > 0.05) (Figure 3a–c). Beta-diversity was used in a comparative analysis of the microbial community composition of different groups. Bacterial beta-diversity was not significantly different among groups (Adonis test: R^2^ = 0.048; *p* = 0.624; Figure 3d).

### 3.4. Differences in Predicted Functions of Gut Microbial

**Figure 4:**
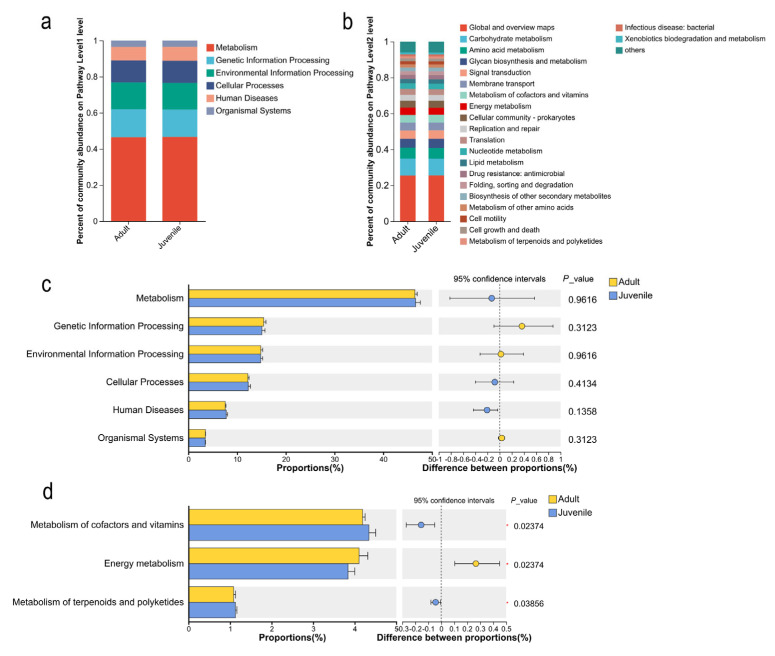
Age difference in cecum microbial KEGG metabolic pathways. (**a**) Composition of KEGG level 1 metabolic pathways. (**b**) Composition of KEGG level 2 metabolic pathways. (**c**) Differential analysis of KEGG level 1 metabolic pathways. (**d**) Differential analysis of KEGG level 2 metabolic pathways. * *p*  < 0.05.

KEGG functional annotation analysis showed that the functional genes of the cecal microorganisms in plateau pikas were divided into six categories in the primary metabolic pathways (Figure 4a). They were mainly enriched in metabolic pathways (46.49% ± 0.46% for adults and 46.62% ± 0.98% for juveniles). However, the abundance did not reach a significant level at different age stages (*p* > 0.05) (Figure 4c). There were 22 metabolic pathways with an abundance greater than 1% in the secondary metabolic pathways (Figure 4b), and there were significant differences in three types of metabolic pathways. Among them, the abundances of metabolism of cofactors and vitamins (4.19% ± 0.02% for adults and 4.34% ± 0.16% for juveniles) and metabolism of terpenoids and polyketides (1.08% ± 0.05% for adults and 1.13% ± 0.03% for juveniles) were significantly higher in the juvenile group than in the adult group (*p* < 0.05), while the abundance of energy metabolism was significantly higher in the adult group than in the juvenile group (4.10% ± 0.21% for adults and 3.84% ± 0.16% for juveniles) (*p* < 0.05) (Figure 4d).

**Figure 5:**
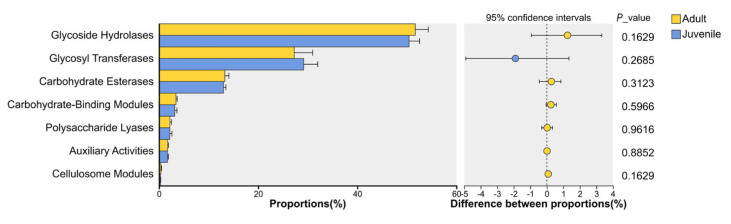
Age difference in cecum microbial CAZy class level.

We used the CAZy database to perform predicted functional annotation of the metabolic-related pathways of the cecum microorganisms in plateau pikas. The order of advantages of the CAZy database annotation was Glycoside Hydrolases (GHs, 51.69% ± 2.62% for adults and 50.44% ± 2.11% for juveniles), Glycosyl Transferases (GTs, 27.29% ± 3.70% for adults and 29.19% ± 2.82% for juveniles), Carbohydrate Esterases (CEs, 13.28% ± 0.81% for adults and 13.01% ± 0.48% for juveniles), Carbohydrate_Binding Modules (CBMs, 3.41% ± 0.23% for adults and 3.16% ± 0.45% for juveniles), Polysaccharide Lyases (PLs, 2.21% ± 0.31% for adults and 2.19% ± 0.43% for juveniles), Auxiliary Activities (AAs, 1.73% ± 0.19% for adults and 1.72% ± 0.16% for juveniles), and Cellulosome Modules (0.38% ± 0.11% for adults and 0.30% ± 0.05% for juveniles). However, the abundance did not reach a significant level at different age stages (*p* > 0.05) (Figure 5).

## 4. Discussion

### 4.1. Differences in Gut Microbiota Among Different Age Groups

We analyzed the gut microbial composition of plateau pikas using metagenomic sequencing. At the phylum level, the gut microbiota was predominantly composed of Bacillota and Bacteroidota. These findings were consistent with previous studies on the gut microbiota of plateau pikas [33,34,53,54], rabbits [55], and mice [56], as well as with research on most mammals [57]. Bacillota primarily function as cellulose degraders [58], breaking down cellulose into short-chain fatty acids to provide energy for the host. Bacteroidete, a key associated microbe, plays a vital role in degrading carbohydrates [59]. Some studies have shown that the abundance of cellulose-degrading bacteria (*Oscillospira* and *Treponema*) in the gut microbiota of plateau pikas decreases, leading to a decline in the ability to degrade and ferment plant-based foods [32]. Some studies have shown that a low-fiber diet reduces the cecal abundance of fiber-degrading bacteria (such as *Prevotella*) [60]. This study found that in the cecal microbiota of juvenile plateau pikas, the proportion of Bacteroidetes increased, while at the genus level, the proportions of Prevotella, Oscillibacter, and Treponema decreased. Therefore, we speculate that juvenile individuals may prefer a low-fiber diet and have a higher demand for easily digestible carbon source foods. Comparable patterns were observed in snails, where juveniles exhibited greater utilization efficiency of proteins and carbohydrates than adults [61].

### 4.2. Age-Mediated Differences in Microbial Diversity

Microbial diversity is essential for ensuring the stability and functional robustness of microbial communities. Alpha diversity is closely related to the overall structure, health, and metabolic capacity of these communities. No significant differences were observed in alpha and beta diversity indices between juvenile and adult plateau pikas. This result might have been due to the small sample size and a large degree of within-group variation, particularly among juveniles, who showed greater variation than adults. A comparable study on captive crab-eating macaques (*Cynomolgus macaques*) also found no significant differences in cecal microbial diversity between age groups [62]. The gut microbiota composition and diversity of adult and juvenile Chinese mystery snails (*Cipangopaludina chinensis*) were also similar, aligning with our findings [61]. However, previous studies on mammals, including wild macaques [63], Brandt’s voles (*Lasiopodomys brandtii*) [64], yellow-bellied marmots [22], and Siberian flying squirrels [21], have reported significant age-related differences in gut microbial diversity, which contrast with our results. Prior studies have also reported that differences in gut microbiota are influenced by dietary differences at different ages [64,65]. Vegetation on the Qinghai–Tibet Plateau is dominated by species typical of alpine meadow [66]. The food resources available to plateau pikas are limited, and captive crab-eating macaques are fed the same food. In contrast, rhesus monkeys, Brandt’s voles, yellow-bellied marmots, and Siberian flying squirrels have abundant food resources. However, diet is not the only factor shaping the gut microbiota; lifestyle, physiological state, and host genetics also influence the composition of the gut microbiota. The specific roles and contributions of these factors in shaping the gut microbiota remain unclear and warrant further investigation.

### 4.3. Predicted Functional Differentiation of Microbiota Across Different Age Stages

The analysis of the composition and differences in KEGG metabolic pathways and CAZY functions of microbial communities between different groups of samples is an effective means of studying the changes in metabolic function that occur in community samples in response to environmental changes. Differential analysis of KEGG level 1 metabolism and CAZy class level showed no significant differences in the overall gut microbial functional profiles between adult and juvenile plateau pikas. Similarly, the gut microbial functional profiles of juvenile and adult Chinese mystery snails exhibited no significant differences [62], which is consistent with the findings of this study. Previous studies have shown that the functional changes in the gut microbiota in plateau pikas are consistent with food nutrition [16]. Therefore, we speculate that there is no significant change in the nutritional requirements or the required nutrient components between juveniles and adult males, but further verification is needed. As is well known, the gut microbiota is closely related to the metabolic capacity of the host. Changes in the composition of gut microbiota may lead to alterations in its function [67]. The KEGG secondary metabolism difference analysis showed that the metabolic abundances of cofactors and vitamins, as well as terpenoids and polyketides, were higher in the juvenile group, while the abundance of energy metabolism was higher in the adult group. We speculate that the proportion of bacteria synthesizing micronutrients and bioactive substances required for growth and development is higher in juvenile individuals, whereas that of bacteria for energy supply is higher in adult male individuals. However, the specific reasons for the differences need to be systematically verified by increasing the sample size, conducting cross-seasonal repeated observations, or combining with the host’s physiological indicators.

### 4.4. Research Limitations and Prospects

Limitations of this study include data collection within a single season, exclusive inclusion of male adult samples, varied ages of juvenile individuals, a relatively small sample size impacting statistical power and generalizability, and data collection during a period of relatively scarce food resources potentially influencing animal physiology and gut microbiota. To address the above issues, future research can be further expanded in the following aspects. Enhance sample collection across seasons and regions for improved temporal and spatial representativeness. Include gender as a variable to analyze its impact on physiological and microbial characteristics. Conduct controlled indoor feeding experiments to study age-related changes in plateau pikas’ gut microbiota. Investigate how seasonal vegetation dynamics affect nutrient intake, digestion, and gut microbiota, and their interaction with age-related variations. Combine multi-season sampling with diet monitoring to better understand environmental and physiological factors affecting plateau pikas’ gut microbiota.

## 5. Conclusions

This study employed metagenomic analysis to reveal the composition and function of the cecal microbial communities in adult and juvenile plateau pikas in different life stages. Overall, there were no significant differences in the gut microbial composition and diversity, KEGG level 1 metabolic pathways, or CAZy class level between adult and juvenile plateau pikas. Despite the similar overall composition, specific age-related changes in taxa and predicted functions were still found. The relative abundance of the core bacterial genus *Oscillospira* was significantly lower in juveniles than in adults. The changes in the proportion of cellulose-degradation-related bacterial communities in juveniles suggest that they tend to choose low-fiber diets. At KEGG level 2 metabolic pathways, the juvenile group had a higher relative abundance of metabolic pathways for cofactors and vitamins, terpenoids, and polyketides, whereas the adult group had a higher relative abundance of energy metabolism. However, this study has limitations, including a single-season sampling design, a small sample size, the use of only male adults, and inaccurate age classification of juveniles. These factors limit the generalizability of the research results. Future work should adopt multi-season, large-scale sampling, include individuals of both sexes, and integrate controlled experiments or host physiological data to verify the observed trends and clarify the main drivers of age-related microbial variation in wild herbivores.

## Figures and Tables

**Table 1:** Core microbiome with 1% relative abundance and present in at least 80% of all samples.

Phylum	Order	Family	Genus	Percentage		Prevalence		*p*-Value
				Adult	Juvenile	Adult	Juvenile	
Bacillota	Eubacteriales	Oscillospiraceae	*Ruminiclostridium*	0.01	0.01	0.90	0.91	1.00
			*Ruminococcus*	0.07	0.07	0.90	0.91	0.23
			*Lawsonibacter*	0.01	0.01	0.90	0.91	0.41
			*Oscillibacter*	0.06	0.04	0.90	0.91	0.04 *
			*Flavonifractor*	0.01	0.01	0.90	0.91	0.47
			*Pseudoflavonifractor*	0.02	0.02	0.90	0.91	0.36
		Clostridiaceae	*Butyricicoccus*	0.03	0.02	0.90	0.91	0.16
			*Clostridium*	0.04	0.04	0.90	0.91	0.96
		Lachnospiraceae	*Roseburia*	0.03	0.03	0.90	0.91	0.96
			*Acetatifactor*	0.07	0.06	0.90	0.91	0.53
			*Blautia*	0.01	0.01	0.90	0.91	0.53
		Eubacteriaceae	*Eubacterium*	0.08	0.07	0.90	0.91	0.67
Bacteroidia	Bacteroidale	Rikenellaceae	*Alistipes*	0.03	0.04	0.90	0.91	0.81
		Prevotellaceae	*Prevotella*	0.13	0.11	0.90	0.91	0.53
		Bacteroidaceae	*Bacteroides*	0.05	0.06	0.90	0.91	0.47
Spirochaetota	Spirochaetales	Treponemataceae	*Treponema*	0.05	0.06	0.90	0.91	0.41
Verrucomicrobiota	Verrucomicrobiales	Akkermansiaceae	*Akkermansia*	0.01	0.01	0.90	0.91	0.96

The * represents a *p*_value less than 0.05.

## Data Availability

The dataset is available on request from the authors.

## Supplementary Materials

The following supporting information can be downloaded at https://www.mdpi.com/article/10.3390/biology15020144/s1; Figure S1: Adult sample contig size distribution; Figure S2: Juvenile sample contig size distribution; Table S1: Sample weight gender table; Table S2: Assembly quality statistics.
