# Supplementary material for: Age-Specific Composition and Predicted Function of Gut Microbiota in Plateau Pikas (Ochotona curzoniae)
Source: Biology (Basel). 2026 Jan 14;15(2):144. doi: 10.3390/biology15020144 (PMC12837580; doi:10.3390/biology15020144)
Supplement: Supplementary file 1 [file biology-15-00144-s001.zip › biology-4038851-supplementary.pdf]

**Table S1**. Sample weight gender table

| Age group | Sample ID | Weight(g) | Sex |
|---|---|---|---|
| Adult | Adult1 | 178.2 | Male |
| | Adult2 | 154.1 | Male |
| | Adult3 | 159.2 | Male |
| | Adult4 | 145.5 | Male |
| | Adult5 | 153.7 | Male |
| | Adult6 | 136.5 | Male |
| | Adult7 | 164.1 | Male |
| | Adult8 | 183.3 | Male |
| | Adult9 | 161.6 | Male |
| Juvenile | Juvenile1 | 28.5 | - |
| | Juvenile2 | 32.8 | - |
| | Juvenile3 | 71.2 | - |
| | Juvenile4 | 63.2 | - |
| | Juvenile5 | 41.0 | - |
| | Juvenile6 | 55.3 | - |
| | Juvenile7 | 66.5 | - |
| | Juvenile8 | 65.7 | - |
| | Juvenile9 | 29.0 | - |

**Table S2** Assembly quality statistics

| Age group | Sample ID | Contigs | Contigs bases(bp) | N50(bp) | N90(bp) | Max(bp) | Min(bp) |
|---|---|---|---|---|---|---|---|
| Adult | Adult1 | 499454 | 318964162 | 647 | 353 | 47055 | 300 |
| | Adult2 | 588643 | 367351537 | 633 | 352 | 214617 | 300 |
| | Adult3 | 304019 | 329667064 | 1749 | 416 | 545729 | 300 |
| | Adult4 | 706211 | 469552974 | 675 | 357 | 115538 | 300 |
| | Adult5 | 292397 | 278592260 | 1285 | 394 | 545738 | 300 |
| | Adult6 | 520033 | 345770187 | 662 | 353 | 228758 | 300 |
| | Adult7 | 402105 | 310683153 | 843 | 368 | 558803 | 300 |
| | Adult8 | 610706 | 370870974 | 609 | 350 | 428699 | 300 |
| | Adult9 | 580895 | 437625241 | 832 | 371 | 326073 | 300 |
| Juvenile | Juvenile1 | 533014 | 344184823 | 664 | 355 | 357618 | 300 |
| | Juvenile2 | 581722 | 413852728 | 749 | 363 | 215272 | 300 |
| | Juvenile3 | 696737 | 428899450 | 620 | 350 | 98018 | 300 |
| | Juvenile4 | 546897 | 362556036 | 679 | 354 | 144227 | 300 |
| | Juvenile5 | 554967 | 398510948 | 755 | 367 | 445928 | 300 |
| | Juvenile6 | 502173 | 378304943 | 813 | 368 | 356867 | 300 |
| | Juvenile7 | 528591 | 403544951 | 839 | 370 | 275887 | 300 |
| | Juvenile8 | 707894 | 503074750 | 739 | 364 | 347339 | 300 |
| | Juvenile9 | 562702 | 415230250 | 760 | 364 | 385687 | 300 |

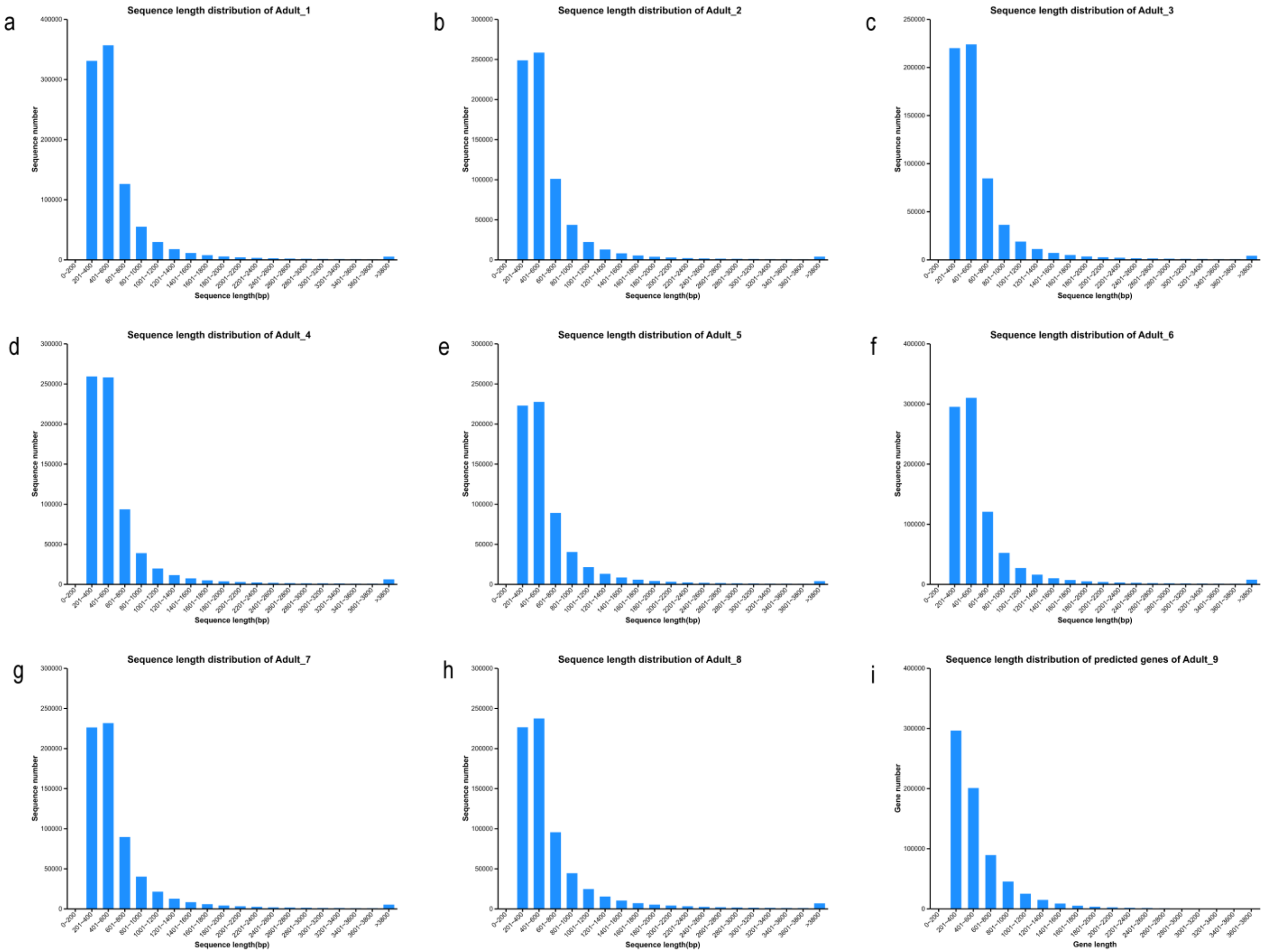


**Figure S1.** Adult sample contig size distribution

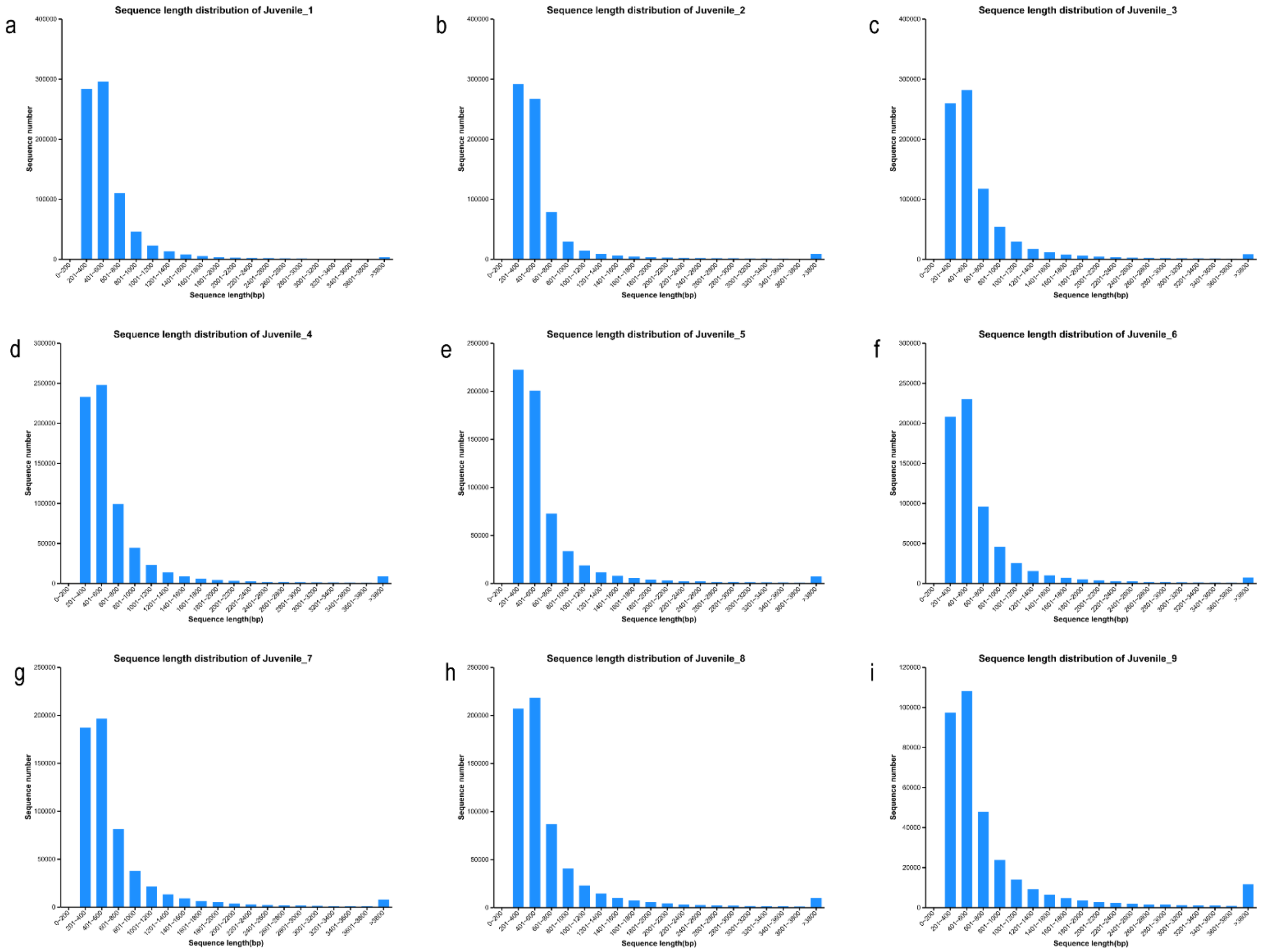


**Figure S2.** Juvenile sample contig size distribution
